# A Study on Detection of Prohibited Items Based on X-Ray Images with Lightweight Model

**DOI:** 10.3390/s25175462

**Published:** 2025-09-03

**Authors:** Tianfen Liang, Hao Wen, Binyu Huang, Nanfeng Zhang, Yanxi Zhang

**Affiliations:** 1 Guangdong Provincial Key Laboratory of Intelligent Port Security Inspection, Huangpu Customs District P.R. China, Guangzhou 510700, China; 2 School of Electromechanical Engineering, Guangdong University of Technology, Guangzhou 510060, China

**Keywords:** prohibited items, X-ray images, deep learning, lightweighting, depthwise separable convolution, dilated convolution spatial pyramid module

## Abstract

X-ray security screening is a well-established technology used in public spaces. The traditional method for detecting prohibited items in X-ray images relies on manual inspection, necessitating security personnel with extensive experience and focused attention to achieve satisfactory detection accuracy. However, the high-intensity and long-duration nature of the work leads to security personnel fatigue, which in turn reduces the accuracy of prohibited items detection and results in false alarms or missed detections. In response to the challenges posed by the coexistence of multiple prohibited items, incomplete identification information due to overlapping items, variable distribution positions in typical scenarios, and the need for portable detection equipment, this study proposes a lightweight automatic detection method for prohibited items. Based on establishment the sample database for prohibited items, a new backbone network with a residual structure and attention mechanism is introduced to form a deep learning algorithm. Additionally, a dilated convolutional spatial pyramid module and a depthwise separable convolution algorithm are added to fuse multi-scale features, to improve the accuracy of prohibited items detection. This study developed a lightweight automatic detection method for prohibited items, and its highest detection rate is 95.59%, which demonstrates a 1.86% mAP improvement over the YOLOv4-tiny baseline with 122 FPS. The study achieved high accurate detection of typical prohibited items, providing support for the assurance of public safety.

## 1. Introduction

As society advances and population mobility increases, public security faces increasingly challenges [1]. The illegal carrying and transportation of prohibited items can easily lead to serious safety accidents, posing a threat to people’s lives and property. Therefore, the development of efficient and accurate detection technologies for prohibited items is particularly important [2,3]. X-ray imaging technology is widely used in the field of prohibited items detection because it can penetrate objects and reveal their internal structures [4,5]. X-ray equipment emits X-rays to penetrate items, and based on the varying degrees of absorption and scattering of X-rays by different substances, it forms a grayscale image to detect potential prohibited items. This technology is widely used in airports, stations, and other public transportation venues, the express logistics industry, government agencies, important facilities, and large event venues for safety inspection purposes. However, traditional X-ray security inspection methods mainly rely on manual identification or apply simple image processing algorithms to assist in judgment, which have issues such as low efficiency, insufficient accuracy, and susceptibility to subjective factors of operator.

In recent years, the rapid development of computer vision and deep learning technologies has advanced the field of prohibited item detection using X-ray imaging [6,7,8,9]. In the early stages of computer vision technology, X-ray images were processed by extracting image information using manual features such as SIFT (Scale-Invariant Feature Transform), SURF (Speeded-Up Robust Features), and HOG (Histogram of Oriented Gradients). These images were classified using SVM (Support Vector Machine) classifiers, and location information was provided by sliding windows [10] or selective search [11]. Franzel T et al. proposed a recognition method using multi-view X-ray images, applying the multi-view integration approach to enhance the recognition of prohibited items’ X-ray images [12]. Bastan M et al. utilized local features and SVM for X-ray image detection, introducing a multi-view search algorithm that employs single-view features to improve recognition accuracy through multiple views [13]. Mery D. implemented adaptive detection with multiple X-ray views, collecting image sequences of objects from various angles, establishing corresponding relationships among different views, and achieving precise detection based on similarity and multi-view information [14]. Mery D et al. also introduced a multi-view detection method, designed to detect the target of interest in a single-view image, followed by matching with multi-view images [15]. Consequently, traditional detection algorithms primarily relied on manually defined features. However, in the context of detecting prohibited items through X-ray images, the complex background and overlapping nature of these images, along with the diverse types of prohibited items, make it challenging for this method to achieve accurate detection.

The deep learning algorithm, which is based on convolutional neural networks, can automatically extract the essential features of detected objects, enabling rapid detection and classification of prohibited items. Convolutional neural network algorithms can be categorized into two-stage object detection algorithms and single-stage object detection algorithms. Two-stage detection algorithms primarily include R-CNN (Region-based Convolutional Neural Networks), Fast R-CNN, and Faster R-CNN [16,17,18], while single-stage detection algorithms encompass YOLO (You Only Look Once) [19] and SSD (Single Shot MultiBox Detector) [20]. The application of convolutional neural networks in X-ray image baggage classification has yielded outstanding results [21]. Consequently, numerous experiments have been conducted by scholars to analyze the performance of classification and object detection in X-ray images [22,23]. Classification involves categorizing images, whereas prohibited items detection requires identifying prohibited items within images and pinpointing their locations. Hence, object detection algorithms are utilized in the detection of prohibited items. Zhang et al. enhanced FSSD (Feature Fusion Single Shot Multibox Detector) [7] by incorporating a semantic enrichment module with dilated convolution to better extract low-level features, and by adding a residual module with residual blocks to FSSD to ensure the extraction of sufficient features. Mu et al. improved upon YOLOv4 by combining dilated convolutions with various dilation rates to expand the receptive field [8]. By incorporating small convolutional asymmetric modules, dilated multi-view convolution modules, and multi-scale feature map fusion strategies, they improved the detection accuracy of multi-scale prohibited items in complex scenarios.

Nevertheless, the detection of prohibited items based on X-ray imaging combined with convolutional neural network algorithms still faces some challenges. These mainly include the complex background of X-ray images, the diverse sizes of prohibited items, and their mutual occlusions and so on, and these characteristics seriously affected the detection accuracy and efficiency of the prohibited items [24,25]. Furthermore, how to further enhance the accuracy and speed of the detection, how to reduce the false alarm rate, and how to improve the portability of the detection equipment are also the current research focuses. In addition, based on our investigation, compared with SSD and FSSD, the YOLO algorithm is relatively simpler and imposes lower hardware requirements, thereby offering notable advantages in environments with limited computational resources. Our contributions in this study are as follows:

(1) A sample set of typical prohibited items is established;
(2) An improved lightweight detection model for typical prohibited items based on the attention mechanism and dilated convolution spatial pyramid module is proposed.

The accuracy of prohibited item detection is improved with the proposed model in this study, which provide support for ensuring public safety.

## 2. Principle and Experimental Setup

### 2.1. X-Ray Imaging Principle

X-rays are electromagnetic waves with high frequency, very short wavelength and great energy. In order to acquire the X-ray image of prohibited items, the radiation source firstly emits X-rays, and then penetrates the object; the X-rays undergoes varying degrees of attenuation. By collecting and digitizing the X-rays with different degrees of attenuation, the digital X-ray images can be formed. The intensity of incident X-rays and the intensity of outgoing X-rays of the prohibited items can be expressed as Equation (1).

(1) $$I=I_{0}e^{-\mu x}$$

In the equation, $I_{0}$ represents the intensity of incident X-rays, I represents the intensity of X-rays after transmission, x is the distance that X-rays travel through the object, and μ represents the attenuation coefficient. The expression of μ is shown in Equation (2).

(2) $$\mu =\sigma \times \frac{N_{\rho }}{A}$$

where σ represents the cross-sectional area of the object through which X-rays penetrate, N represents Avogadro’s constant, ρ represents the density of the object, and A represents the molar mass of the substance.

### 2.2. Analysis of Prohibited Items on X-Ray Images and Enhancement of Datasets

Prohibited items may be present in any part of the luggage, and multiple prohibited items may be stacked together. Therefore, during X-ray imaging, the outlines of each prohibited item are prone to overlap with each other. Meanwhile, only the outline of the inspected object can be obtained by X-ray imaging, while the detailed features such as the texture and color of the prohibited items cannot be captured by X-ray imaging. This poses a challenge for the effective detection of these prohibited items. Furthermore, the types of prohibited items are numerous, making it difficult to collect the X-ray imaging results of all prohibited items in various situations. In order to improve the detection accuracy, it is necessary to continuously expand the sample set of prohibited items that has been obtained, and to acquire the diverse features of the prohibited items.

**Figure 1:**
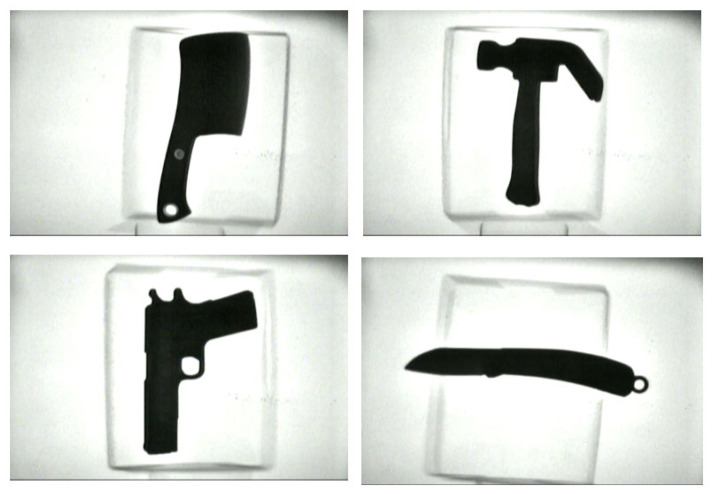
X-ray images with a single typical prohibited item and a simple background.

Based on actual security inspection scenarios, this study employs X-ray machines, which are operated within the safe radiation range, to collect the X-ray images of the typical prohibited items and create a sample set. The sample set includes X-ray image samples of 8 types of objects such as pistols, kitchen knives, scissors, knives, screwdrivers, wrenches, hammers, and lighters. Firstly, various types of prohibited items were collected in simple backgrounds, as shown in Figure 1. In these images, there were no other items and no occlusions between objects. The captured images could clearly display the complete contour information of various prohibited items. For such images, the categories of various prohibited items can also be easily distinguished by human eyes. Thus, it is considered that these images clearly and completely captured the characteristics of various prohibited items, which is conducive to obtaining the characteristics of the typical prohibited items by these X-ray images.

**Figure 2:**
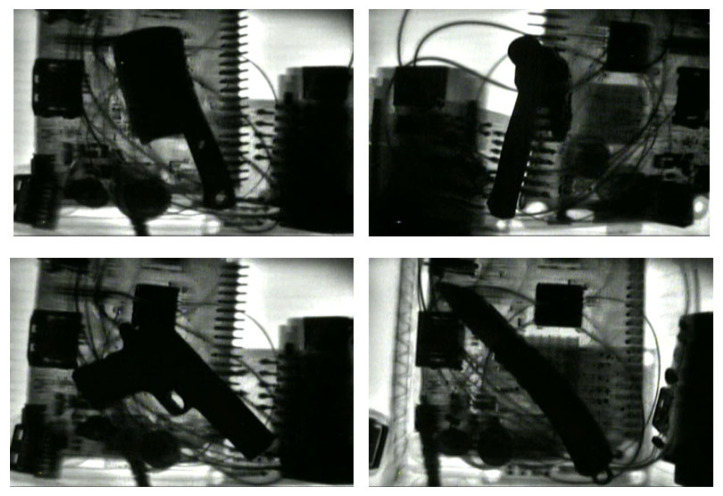
X-ray images of a single prohibited item on a complex background.

In addition to these simple background X-ray images, the dataset also includes images with a single typical prohibited item and complex backgrounds, as shown in Figure 2. In these images, the background is more complex, but it contains only one type of prohibited item, without multiple prohibited items overlapping each other. Due to the complex imaging background, the prohibited items overlap and are occluded by the miscellaneous objects, and the contour information of the prohibited items is disordered and incomplete, which affects the detection accuracy of the prohibited items.

**Figure 3:**
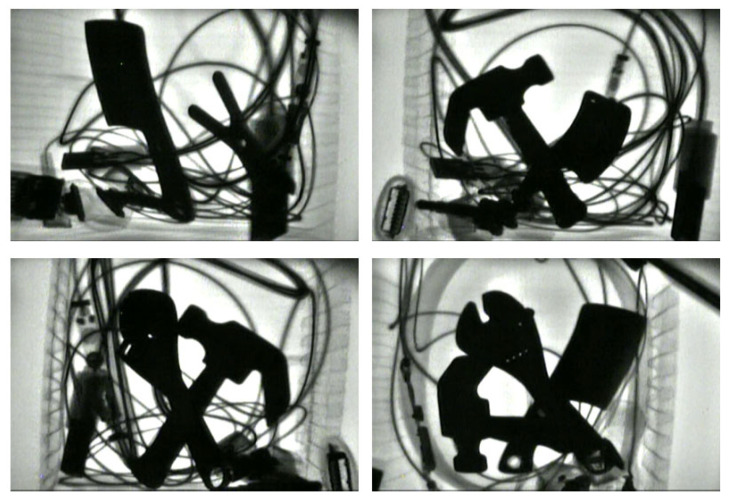
X-ray images of multiple prohibited items with complex background.

A typical scenario is that multiple prohibited items appear simultaneously and are piled up haphazardly together. This study also fully considered images of such scenarios in the establishment of the sample set of the typical prohibited items, as shown in Figure 3. In such scenarios, the image background is complex, and multiple prohibited items appear simultaneously, and most of them have mutual occlusion.

**Figure 4:**
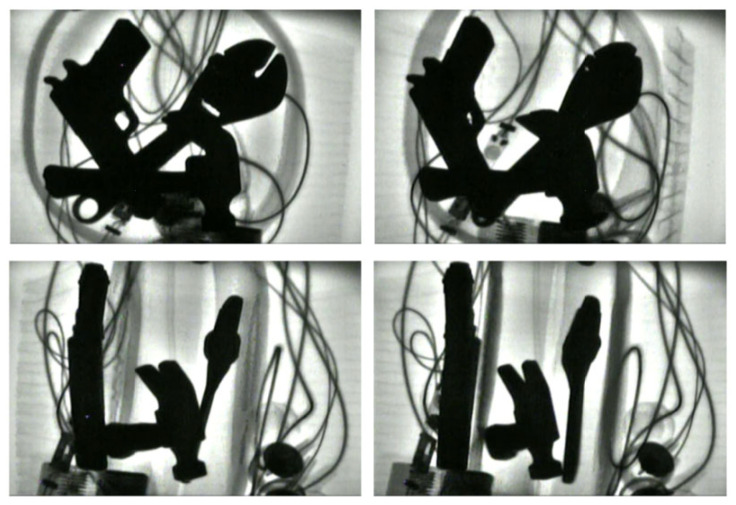
X-ray images with multiple prohibited items from different angles and complex background.

To acquire the diversity of the prohibited items sample set, this study also collected X-ray images of prohibited items from different imaging angles, such as 0° (top-down), 30°, 45°, and 60° relative to the transport plane, as shown in Figure 4. The characteristic information of prohibited items is suitable prohibited items are complex, and hard to be detected. Furthermore, there are varying degrees of obstruction between multiple prohibited items. All these obstacles pose challenges for detection of prohibited items.

In order to enhance the robustness of the model proposed in this study, the sample set is also expanded through image flipping, blurring, and affine transformation, thereby to simulate the robust characteristics of the typical prohibited items in the actual scenarios.

#### 2.2.1. Image Flipping of X-Ray Imaging of Prohibited Items

**Figure 5:**
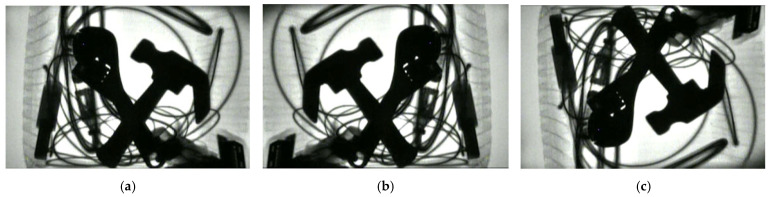
Image flipping of the X-ray image of prohibited items. (**a**) The original X-ray images; (**b**) horizontal flipping; (**c**) vertical flipping.

In reality, the X-ray imaging directions of the prohibited items are various; therefore, image samples collected in only one direction cannot simulate the real scenario. By performing multi-directional image flipping of the collected X-ray images, the sample sets of the overlapping situation of typical prohibited items can be expanded with multiple perspectives. Image flipping is usually divided into horizontal flipping and vertical flipping, as shown in Figure 5. The application of the image flipping increases the diversity of the sample set.

#### 2.2.2. Image Blurring of the X-Ray Images of Prohibited Items

**Figure 6:**
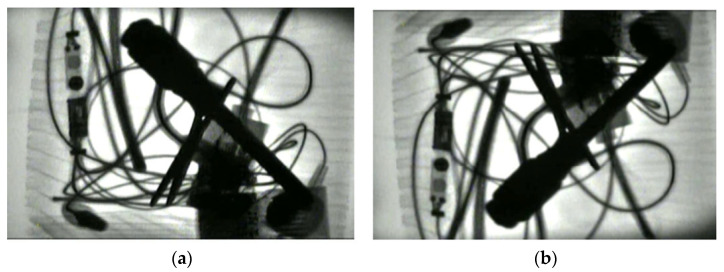

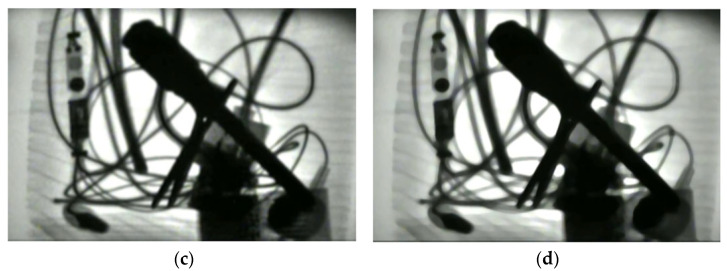
Image fuzzy of prohibited items X-ray image. (**a**) The original captured X-ray image; (**b**) The image with Gaussian blurring; (**c**) the image with averaging blurring; (**d**) the image with median blurring.

Image blurring is the operation of blurring the X-ray images by Gaussian blur, averaging blur and median blur techniques. In most cases, the images of prohibited items produced in the laboratory are good quality and do not show blurring. However, in the real detection scenarios, due to the movement of the conveyor belt or other external vibrations, X-ray images may be blurred. By applying image blurring techniques, it is possible to simulate real detection scenarios. In this study, the standard deviation used for Gaussian blurring ranges from 0 to 3, and the larger the value, the greater the intensity of the blurring effects accomplished. The size of the averaging blurring filter window is set to 2–7, and the larger the filter window, the stronger the blurring effect accomplished. The median blurring filter window is set to 3–11, and the filtering effect increases as the filter window size increases. The blurred effects of the X-ray images of the prohibited item are shown in Figure 6.

#### 2.2.3. Affine Transformation of X-Ray Images of Prohibited Items

**Figure 7:**
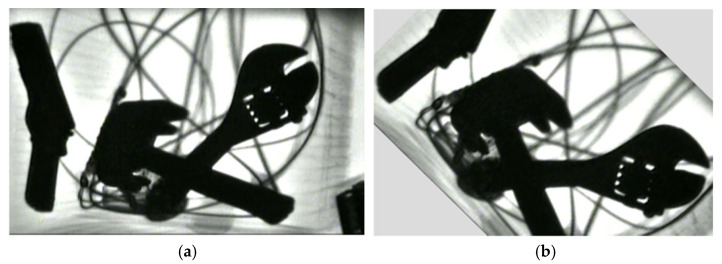
Affine transformation of prohibited items X-ray image. (**a**) The original captured X-ray image; (**b**) the affine transformation of (**a**).

Affine transformations of images include image scaling, translation, rotation, and other processing operations. The image scaling magnifies the X-ray images of the prohibited item by 0.8 to 1.2 times, to simulate the imaging effect of the object at different distances, in order to obtain the characteristics of the prohibited item at multiple scales. Image translation is achieved by shifting the original image by a positive or negative 20% of its position. Image rotation is achieved by rotating the original image by either +45 degrees or −45 degrees. The above-mentioned several transformations are randomly combined to generate new X-ray images of prohibited items. The newly generated images have a different ratio compared to the original images, thereby enabling the sample set of prohibited items to achieve greater differences from operations such as image flipping and image blurring. In affine transformation, the size, position and orientation of the prohibited items all change in different styles, effectively increasing the diversity of the prohibited items sample set. The affine transformation of the prohibited item’s X-ray images are shown in Figure 7.

#### 2.2.4. Mosaic Transformation

In the target detection task, the Mosaic data augmentation technique generates diverse training samples by randomly combining four images and applying operations such as scaling and cropping. In our study, the Mosaic data augmentation operation randomly combines four sample images into a new sample image, which can increase the diversity of the sample set and greatly enrich the background of the samples, thereby improving the generalization ability of the proposed model.

## 3. The Proposed Model and Its Training

### 3.1. The Structure of the Proposed Model

YOLOv4-tiny has significantly reduced the CSPDarknet53 backbone network which is applied in YOLO, decreasing its number of convolutional layers and channels to enhance the algorithm’s speed, making it more suitable for deployment on portable platforms. The backbone network of YOLOv4-tiny consists of convolutional modules and residual modules. To be specific, the backbone network of YOLOv4-tiny consists of two convolutional layers and three residual modules and uses the computationally efficient LeakyReLU as its activation function. However, this lightweight design involves inherent trade-offs. While it attains a processing speed of 168 FPS, the simplified architecture lacks specialized modules for addressing occluded objects and demonstrates reduced effectiveness in detecting multi-scale items within complex and cluttered environments.

This study removed the channel division operation and replaced the 3 × 3 conventional convolution in the original module with a depthwise separable convolution (DSC). DSC firstly performs a convolution operation on each channel of the input feature map, and then uses a 1 × 1 convolution to adjust the number of channels of the feature map. By adopting DSC, the number of parameters in the convolution process is reduced, thereby lowering the computational complexity of the convolutional process. Instead of processing all image details simultaneously, it first analyzes basic shapes in separate channels then combines key patterns. This reduces model size by 17% while preserving detection capability, crucial for portable scanners.

Meanwhile, the proposed model incorporates an SE (Squeeze-and-Excitation Networks) attention module before the residual module outputs the features, to calculate the weight of the feature values extracted from different channels. Through this mechanism, the proposed model can dynamically adjust the weights of different channels and enhance the model’s expression ability, while retaining the original features. The SE attention mechanism consists of two operations: compression and activation. The compression operation involves global average pooling on the input features, thereby producing a better acquisition of the global information of the features. Global average pooling reduces the feature map with dimensions *H* × *W* × *C* to a feature map of 1 × 1 × *C*, that is, it compresses the global spatial information into a one-dimensional vector. The calculation equation of global average pooling is shown in Equation (3), where $z_{c}$ represents the results of the compression; H,W and *C* represent the Height, Width and Channels of the input map $u_{c}$ respectively.

(3) $$z_{c}=\frac{1}{H\times W}\sum_{i=1}^{H}\sum_{j=1}^{W}u_{c}(i,j)$$

The activation operation consists of two fully connected layers. The first fully connected layer transforms the result of global pooling into a vector of C/r dimensions, and then applies the ReLU activation function. The second fully connected layer restores the C/r-dimensional vectors to C-dimensional vectors, and then applies the sigmoid activation function, thereby obtaining the weight matrix S. Its calculation equation is shown in Equation (4), where δ is the ReLU activation function, and σ is the sigmoid activation function. *s* represents result of the activation operation.

(4) $$s=\sigma (\delta (z_{c}))$$

**Figure 8:**
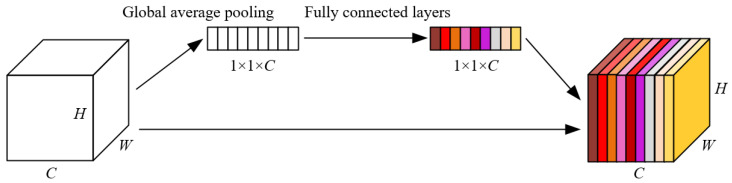
The structure of the SE module.

The SE attention module does not alter the dimension and scale of the features. Its module structure diagram is shown in Figure 8.

In this study, the DSC module, batch normalization, LeakyReLU activation function and SE attention module were combined to form an new residual module, marked as Dw_Resblock_SE_body. In addition to adding the SE attention module to the backbone network, this study also added the SE attention module before its two output heads, to enhance the quantization of different channels, and improve the detection accuracy of prohibited items.

**Figure 9:**
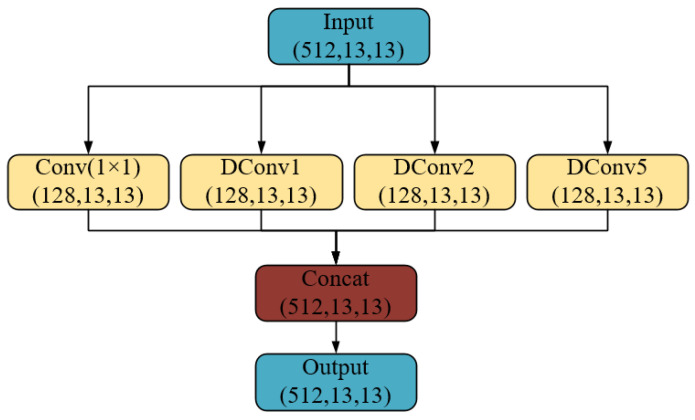
Structure of DCSP.

Meanwhile, this study adds a Dilated Convolution Spatial Pyramid module (DCSP) after the feature layer of the backbone network, to extract multi-scale features of prohibited items. The dilated convolution, compared to the traditional convolution, incorporates the expansion rate as an additional parameter. Adjusting the expansion rate leads to the acquisition of a larger receptive field with the same number of parameters. In the dilated convolution spatial pyramid module, 1 × 1 convolutions and 3 × 3 convolutions with three dilation rates of 1, 2, and 5 are used to obtain different scales of receptive fields, as shown in Figure 9. Therefore, there are four types of receptive fields, namely the 1 × 1 receptive field obtained from the 1 × 1 convolution, as well as the 3 × 3, 5 × 5, and 11 × 11 receptive fields obtained by combining the 3 × 3 convolution with different dilation rates. To reduce the computational cost of the algorithm, the number of output feature-map channels by each convolution operation is reduced to 1/4 of the number of the input feature-map channels. Finally, the four output feature maps are stacked together along the channel dimension, so that the feature-map channels after passing through the DCSP are consistent with the number of channels before this module.

**Figure 10:**
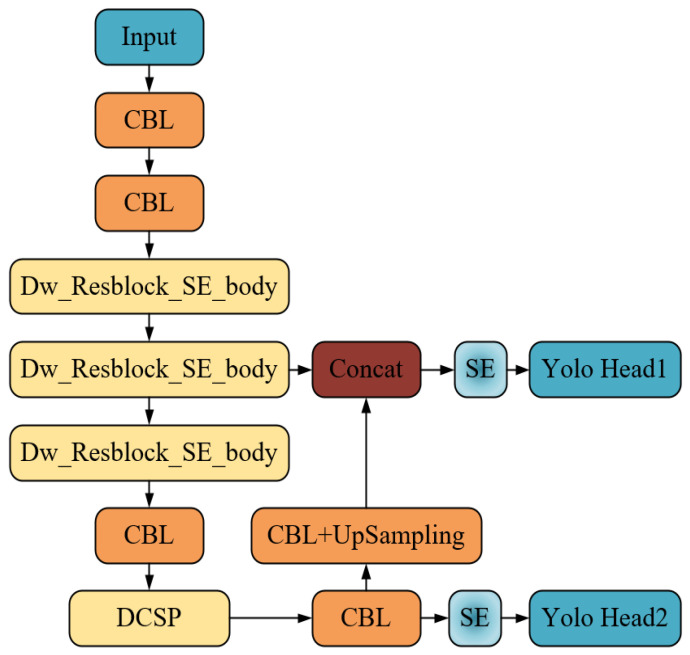
The overall framework of the proposed DW-SE-DCSP-YOLO.

The overall framework of the improved model in this study named DW-SE-DCSP-YOLO, is shown in Figure 10. The CBL represents the combination of the convolution, batch normalization and LeakyReLU activation function.

### 3.2. Training of the Proposed Model

Considering the complexity of the X-ray images of prohibited items, this study established a sample set including 8 typical prohibited items, and their X-ray images were obtained under different angles, different backgrounds, and different degrees of occlusion. The sample set is enhanced by the methods mentioned in Section 2.2, and it includes 5015 images acquired by X-ray machines or generated by enhancement of the dataset in total. In these images, there are 8413 prohibited items are captured, which are 1186 pistols, 1717 kitchen knives, 804 scissors, 820 small knives, 899 screwdrivers, 1042 wrenches, 1046 hammers, and 899 lighters.

As shown in Figure 10, the proposed model firstly extracts features from the X-ray images of the typical prohibited items. After several operations including convolution, residual blocks, pooling, and attention modules, two different-scale feature maps are output, with the scales of (256, 26, 26) and (512, 13, 13), respectively. Secondly, the feature map with scales (512, 13, 13) is transmitted to the newly added DCSP. Based on the multi-scale features of prohibited items obtained by the DCSP, the feature maps are further processed by one more convolution operation, and then transmitted to two following branches. One branch is the SE attention mechanism module, and the output feature map with a scale of (39, 13, 13) is produced by a Yolo Head module; the other branch undergoes upsampling operation to obtain a feature map with a scale of (128, 26, 26), and then this map is stacked with the feature map of scale (256, 26, 26) in the channel dimension, thereby forming a feature map of scale (384, 26, 26). The feature map of scale (384, 26, 26) is further processed by another SE attention mechanism module and the Yolo Head module, resulting in an output feature map with a scale of (39, 26, 26). Therefore, the Dw-SE-DCSP-YOLO model proposed in this study obtained two different-scale output feature maps, with scales of (39, 13, 13) and (39, 26, 26), respectively. This enables the proposed models can acquire the features of the prohibited items in different channels and at different scales, thereby enhancing its detection capability of prohibited items based on X-ray images.

In this study, the training operating system is Windows 10. The CPU is AMD Ryzen 3600, and its memory is 16 GB. The graphics card is NVIDIA GeForce GTX 1080Ti, and its memory is 11 GB. The deep learning framework is deployed with PyTorch 2.0. The optimizer is SGD. The number of training batches is set as 32. The initial learning rate of the model established is set as 0.001. The sample set is divided by a 7:3 ratio, and 70% samples form the training set, and 30% samples form the testing set. The actual quantities of prohibited items contained in the training set and the test set are shown in Table 1.

## 4. Result and Analysis

The detection of prohibited items is not only expected to determine the category of the prohibited items, but also to locate the prohibited items in the X-ray images. Therefore, considering only the detection rate of prohibited items as the criterion for evaluating the performance of the model is not comprehensive. The target detection algorithms usually employ precision (P) and recall (R) as evaluation standard. P is also known as the “true positive rate”, which represents the proportion of the predicted positive samples that are actually positive; R indicates the proportion of the positive samples that are correctly predicted. The expressions of P and Rare shown in Equations (5) and (6), respectively.

(5) $$P=\frac{TP}{TP+FP}$$

(6) $$R\;=\frac{TP}{TP+FN}$$

where TP represents the number of true positive samples that were correctly predicted, FP represents the number of true positive samples that were incorrectly predicted, and FN represents the number of true negative samples that were incorrectly predicted.

In this study, the F-Measure, which is the weighted harmonic mean of P and R, is employed as an indicator to evaluate the performance of the established model, and its expression is shown in Equation (7).

(7) $$F=\frac{(a^{2}+1)PR}{P+R}$$

where a is a variable parameter, and a is usually set to 1.

*AP* (Average Precision) is another commonly used performance evaluation metric in object detection, and it is also a comprehensive evaluation standard that combines precision and recall rates. The *AP* is obtained by calculating the area under the *P*(*R*) curve, which is the area of the function relationship between precision and recall within the range of [0, 1]. The expression of *AP* is shown in Equation (8).

(8) $$AP=\int_{0}^{1}P\left(R\right)dR$$

By averaging all the *AP* values of each category, the *mAP* standard can be obtained. Its calculation is shown in Equation (9), where *n* denotes the number of categories.

(9) $$mAP=\frac{1}{n}\sum_{i=1}^{n}AP_{i}$$

**Figure 11:**
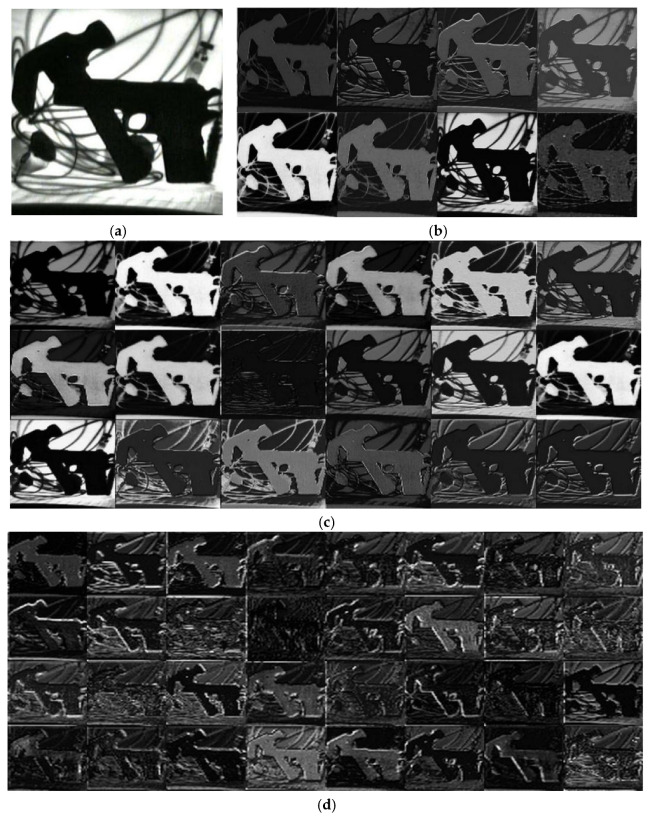

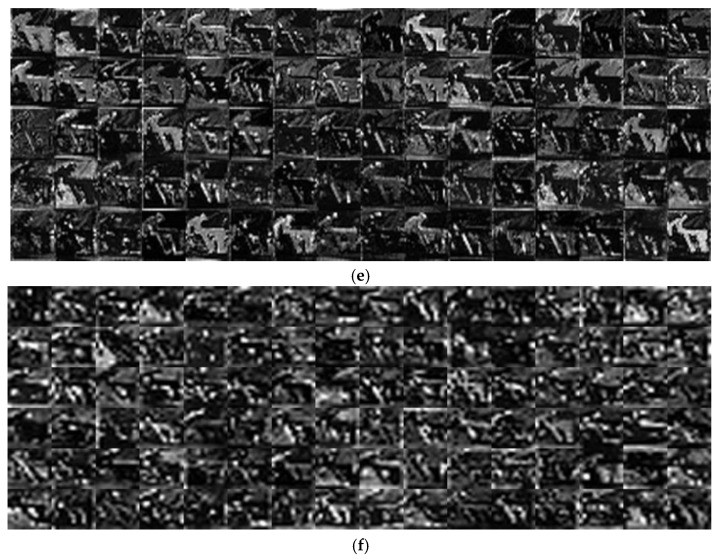
The visualization of the feature map during the process of the proposed model. (**a**) The original X-ray images of a sample; (**b**) The visualization of its feature map after the first convolution operation; (**c**) The visualization of its feature map after the second convolution operation; (**d**) The visualization of its feature map after the first Dw_Resblock_SE_body module; (**e**) The visualization of its feature map after the second Dw_Resblock_SE_body module; (**f**) The visualization of its feature map after the third residual convolution module.

By obtaining the feature maps of typical prohibited items during the model processing, the extraction ability of the model established in this study for the characteristics of prohibited items can be demonstrated and visualized, shown in Figure 11. Figure 11a shows the X-ray images of a hammer and a pistol. Figure 11b presents the visualization of the feature map after the first convolution operation of Figure 11a. Figure 11c shows the visualization of the feature map after the second convolution operation of Figure 11a. Figure 11d–f represent the visualization of the feature map after the operations of the first, second, and third residual modules of Figure 11a, respectively. Due to the fact that the original X-ray images contain relatively little detailed texture information, the outline information of the prohibited items is more prominently displayed in the visualized feature map. After multiple convolutions, the information of the prohibited item’s feature map gradually transforms from clear contour information to its essential feature data.

**Figure 12:**
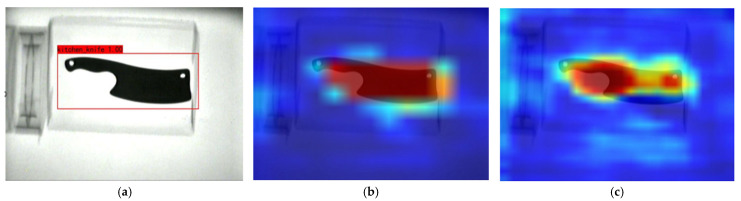
Improved YOLOv4-tiny heat maps for prohibited items recognition. (**a**) Detection of the prohibited items; (**b**) Heat map with scale (13, 13); (**c**) Heat map with scale (26, 26).

The heat map of the prohibited items during the detection by the proposed Dw-SE-DCSP-YOLO in this study is shown in Figure 12. It is shown that the prohibited items were successfully detected in Figure 12a. Figure 12b shows the heat map of the feature map with a scale of 13 × 13. The darker the red color, the greater weight of the feature extracted from that area. Figure 12c shows the heat map extracted from a feature map with a scale of 26 × 26. Considering the color of the feature, the detected target’s weight is lower compared to that in Figure 12b, indicating the difference in the weights of feature values for the same target under different scales.

**Figure 13:**
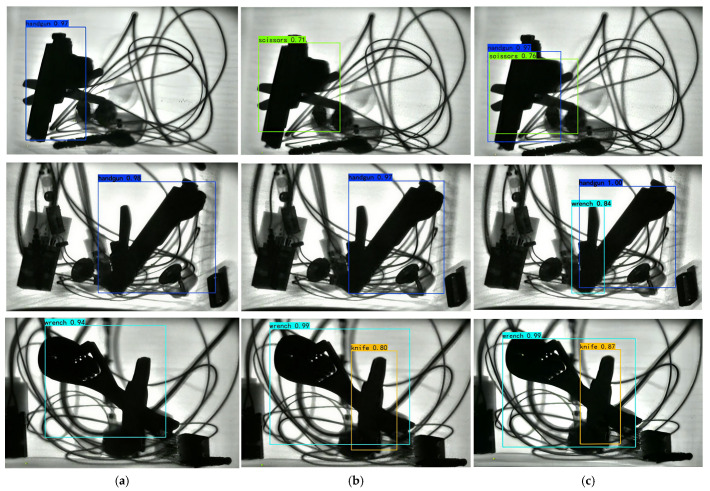
Comparison of recognitions of each prohibited items recognition algorithm. (**a**) The detection effect of YOLO v3-tiny; (**b**) the detection effect of YOLO v4-tiny; (**c**) the detection effect of our proposed model.

**Figure 14:**
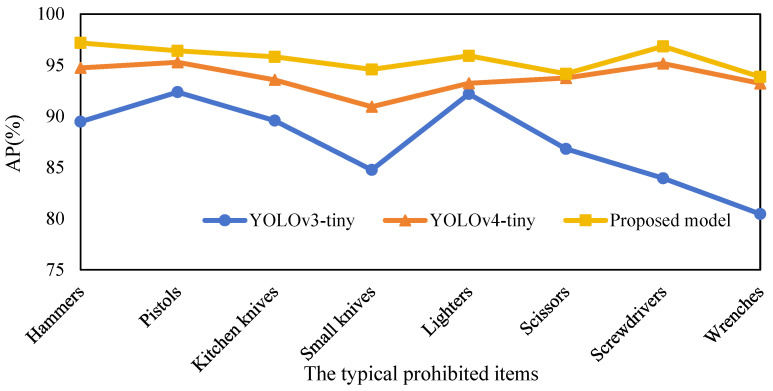
AP values of each prohibited items recognition algorithm.

To verify the effectiveness of the proposed Dw-SE-DCSP-YOLO algorithm, a comparative experiment was conducted with YOLOv3-tiny and YOLOv4-tiny, shown in Figure 13. The detection AP of each algorithm is shown in Table 2. Comparison of experimental results of various detection algorithms for prohibited items. In Figure 14, the detection accuracies of these three algorithms for the 8 typical prohibited items all exceed 80%, and the detection AP of the Dw-SE-DCSP-YOLO algorithm are all higher than 93%, and its lowest value is for Wrench, with an AP of 93.86%, and its highest value is the Hammer, with an AP of 97.17%. Moreover, the detection AP of Dw-SE-DCSP-YOLO for all types of prohibited items are very even, indicating the capability of its detection of different types of prohibited items are nearly the same.

To verify the effectiveness of the improved module in this study, an ablation test was conducted. The test results are shown in Table 3. The recognition effect of algorithms with different modules for prohibited items. In Table 3, A, B, and C represent the Dw_Resblock_SE_body module, DCSP and SE module, respectively. In Table 3, the model has an mAP increase of 0.52% compared to the original YOLOv4-tiny. This indicates that improving the backbone network is beneficial for enhancing its ability to extract features of contraband items. By adding an attention module before the output head of YOLOv4-tiny, its mAP was improved by 1.13% compared to the YOLOv4-tiny model, indicating that the attention module is also effective. The above results indicate that the three improvement measures proposed in this study can enhance the detection performance of the algorithm for prohibited items in X-ray images. Among them, the visible void convolution spatial pyramid module is the most effective among the three improvements.

The application of Mosaic dataset enhancement technology can effectively increase the diversity of samples and enhance the detection performance of the established model in scenarios where multiple prohibited items coexist or are mutually occluded. Performance comparison of algorithms with different modules for prohibited items is shown in Table 4, After conducting ablation tests on modules A, B, and C, the detection accuracy of the model using Mosaic has improved. In Table 4, the mAP of YOLOv4-tiny without using Mosaic was 91.83%. The improvements have all enhanced recognition accuracy. Specifically, after adopting the improved backbone network, the mAP increased by 1.25%; after adding the dilated convolution spatial pyramid module, the mAP increased by 2.05%; and after adding the attention mechanism before the output head, the mAP increased by 1.21%. When all the above three improvement measures are added to the network, the detection effect is the best, with an improvement of 2.6% in mAP compared to YOLOv4-tiny. As can be seen from Table 4, the mAP of YOLOv4-tiny increased by 1.9% by applying Mosaic. The improved algorithm also saw an increase of 1.16% in mAP after being trained with Mosaic. In terms of computing speed, after the respective addition of each of the above modules, the detection speed of the model has, but it still remains above 120 FPS, which does not affect the rapid detection of prohibited items based on X-ray images. Furthermore, as can be seen from Table 4, the size of the model with the addition of Module A is smaller than that of the YOLOv4-tiny. This is because the DSC in Module A reduces the parameters of the conventional convolution operation, thereby saving space. Since the B model incorporates multiple convolution operations, it occupies the largest amount of space; while the added SE attention module has a relatively small code volume, thus the spatial size of the C model remains largely unchanged.

## 5. Conclusions

This study firstly improved the residual module, replacing the traditional convolution operation with DSC to reduce the size of the algorithm. Secondly, based on the aforementioned improvements, this study added an attention module to the model, enhancing its feature extraction capability. Additionally, an attention module was further added before the network’s output layer to improve the channel attention of the algorithm. Finally, a dilated convolutional spatial pyramid module was added after the backbone network. Different dilation rates of dilated convolutions were used to extract multi-scale features of prohibited items from the samples and perform feature fusion. This increased the model’s ability to extract multi-scale features and achieved lightweight and rapid detection of various dangerous items in complex backgrounds. The experiment shows that, DW-SE-DCSP-YOLO demonstrates a 1.86% mAP improvement from 93.73% to 95.59% over the YOLOv4-tiny baseline while maintaining real-time performance at 122 FPS. Our proposed model is able to be deployed on portable equipment with less computation resources and capabilities and effectively meet the detection requirements for prohibited items in complex X-ray security scenarios.

In the future, an enhanced learning algorithm is expected to be incorporated into the model. We will benchmark DW-SE-DCSP-YOLO on established public datasets such as SIXray against prevailing lightweight detectors—including MobileNet-SSD, EfficientDet-Lite and common YOLO variants—to further validate its generalizability and sharpen its competitive edge. With the increase in the number of detection samples in actual deployment, the accuracy of detecting prohibited items is also expected to be continuously improved.

## Figures and Tables

**Table 1:** Types of prohibited items and the training sets and test sets for model training.

Sample Set	Hammers	Pistols	Kitchen Knives	Small Knives	Lighters	Scissors	Screwdrivers	Wrenches	Total
Training Set	722	801	1217	580	628	571	647	725	5891
Testing Set	324	385	500	240	271	233	252	317	2522
Total number	1046	1186	1717	820	899	804	899	1042	8413

**Table 2:** Comparison of experimental results of various detection algorithms for prohibited items.

Algorithms	Hammers	Pistols	Kitchen Knives	Small Knives	Lighters	Scissors	Screwdrivers	Wrenches	mAP
YOLOv3-tiny	89.47	92.37	89.58	84.76	92.19	86.82	83.95	80.46	87.45
YOLOv4-tiny	94.73	95.28	93.56	90.94	93.23	93.74	95.16	93.21	93.73
Proposed model	97.17	96.4	95.81	94.58	95.91	94.15	96.84	93.86	95.59

**Table 3:** The recognition effect of algorithms with different modules for prohibited items.

Algorithms	Hammers	Pistols	Kitchen Knives	Small Knives	Lighters	Scissors	Screwdrivers	Wrenches	mAP
YOLOv4-tiny	94.73	95.28	93.56	90.94	93.23	93.74	95.16	93.21	93.73
YOLOv4-tiny + A	96.22	96.28	95.27	91.77	95.49	91.76	94.68	92.52	94.25
YOLOv4-tiny + B	97.19	96.7	95.48	93.36	95.42	95.5	94.9	93.51	95.26
YOLOv4-tiny + C	96.56	96.77	94.57	93.59	95.89	92.25	95.48	93.8	94.86
Proposed model	97.17	96.4	95.81	94.58	95.91	94.15	96.84	93.86	95.59

**Table 4:** Performance comparison of algorithms with different modules for prohibited items.

Algorithms	mAP	mAP	FPS	Storage Space (MB)
	No Mosaic	Mosaic		
YOLOv4-tiny	91.83	93.73	168	23
YOLOv4-tiny + A	93.08	94.25	142	19
YOLOv4-tiny + B	93.88	95.26	131	30
YOLOv4-tiny + C	93.04	94.86	152	23
Proposed Model	94.43	95.59	122	27

## Data Availability

Data will be made available on request.
